# The *int22h1/int22h2*-Mediated Xq28 Duplication Syndrome: An Intersection between Neurodevelopment, Immunology, and Cancer

**DOI:** 10.3390/genes12060860

**Published:** 2021-06-04

**Authors:** Rami A. Ballout, Ayman W. El-Hattab

**Affiliations:** 1 Lipoprotein Metabolism Section, Translational Vascular Medicine Branch, National Heart, Lung and Blood Institute (NHLBI), National Institutes of Health (NIH), Bethesda, MD 20814, USA; 2 Department of Clinical Sciences, College of Medicine, University of Sharjah, Sharjah P. O. Box 27272, United Arab Emirates; 3 Clinical Genetics, University Hospital Sharjah, Sharjah P. O. Box 72772, United Arab Emirates

**Keywords:** XLID, gene dosage, RAB39B, CLIC2, BRCC3, VBP1, MECP2

## Abstract

The *int22h1/int22h2*-mediated Xq28 duplication syndrome is a rare X-linked intellectual disability syndrome (XLIDS) arising from a duplication of the segment between intron 22 homologous regions 1 and 2, on the q28 subregion of the X chromosome. The main clinical features of the syndrome include intellectual disability, neurobehavioral abnormalities, and dysmorphic facial features. Due to the X-linked nature of the syndrome, affected males exhibit more severe phenotypes compared with heterozygous females. A unique distinguishing feature of the syndrome across the sexes, however, is a peculiar combination of recurrent sinopulmonary infections and atopy exclusively seen in a subset of affected males. In addition to the ‘typical’ 0.5 Mb duplication detected in most cases reported to date with the syndrome, a shortened centromeric version, and another 0.2 Mb telomerically shifted one, have been recently identified, with most detected duplications being maternally inherited, except for three recent cases found to have de novo duplications. Interestingly, a recently reported case of an affected male suggests a possible association of the syndrome with multiple malignancies, an observation that has been recently replicated in two pediatric patients. As a result, a better understanding of the pathogenesis of *int22h1/int22h2*-mediated Xq28 duplication syndrome may grant us a better understanding of the sex-specific differences in immunological responses, as well as the potential role of the genes involved by the duplication, in oncogenesis.

## 1. Background

To date, more than 140 genes located on the X chromosome have been associated with distinct, inherited forms of intellectual disability, collectively termed X-linked intellectual disability syndromes (XLIDS) [1].

Clinically, XLIDS are classified as syndromic or non-syndromic [1,2,3]. Individuals with syndromic XLIDS generally present with a constellation of physical and/or neuropsychiatric features, in addition to their intellectual disability. In contrast, individuals with non-syndromic forms of XLIDS present either with isolated intellectual disability, or a combination of intellectual disability and inconsistent or nonspecific additional findings [2,3].

Interestingly, a large subset of the genes associated with distinct XLIDS falls within the q28 region of the X chromosome, with dosage alterations in these genes, i.e., duplications or deletions, being the most common genetic alterations underlying the different XLIDS [4,5]. Of the numerous XLIDS arising from gene dosage alterations in the Xq28 region, *MECP2* dosage alterations are probably the most known or heard of within the medical community, since deletions and duplications involving the *MECP2* locus have been associated with two distinct forms of syndromic XLIDS having multiple overlapping features: Rett syndrome and *MECP2* duplication syndrome, respectively [5,6,7]. Nonetheless, while the specific phenotypic features of the distinct types of Xq28 duplication syndromes (OMIM # 300815) vary depending on the specific genes within the Xq28 region that are affected by the duplication [5,8], all Xq28 duplication syndromes tend to affect multiple organ systems due to the remarkably high gene density of that region [8,9].

A common feature across the various XLIDS, however, is their attenuated phenotypic manifestations in heterozygous females, compared with affected males. This is not surprising since the latter are hemizygous, while the former are heterozygous for the genetic changes underlying these syndromes [10,11,12]. Additionally, the X-inactivation process that occurs in females, also plays a key role in the final manifesting phenotype, since many heterozygous females undergo skewed (i.e., selective) inactivation of the X-chromosome harboring a genetic abnormality, further attenuating the corresponding phenotypic findings [11,13].

Among the various Xq28 duplication syndromes identified to date, the *int22h1/int22h2*-mediated Xq28 duplication syndrome may be regarded as one of the relatively recently discovered XLIDS, since it was first described in 2011 [14]. To date, a total of 35 cases have been reported within the literature, since the first description of this syndrome, granting it the designation of an ultra-rare disorder (i.e., global prevalence < 1 in 50,000 individuals) [15,16]. Nonetheless, the uniqueness of this syndrome that warrants dedicating this review for its discussion, lies in its constitution of a potential intersection point in the investigation and ultimate understanding of the sex-specific differences in neurodevelopment, immune system responsiveness, and cancer development. As such, apart from summarizing the phenotypic features reported to date in association with the *int22h1/int22h2*-mediated Xq28 duplication syndrome, this review sheds light on the hypothetical roles of the different genes contained within the duplicated segment, in the development of specific manifestations of the syndrome, in the hope that this review prompts the interest of the scientific community in further characterizing this fascinating syndrome.

## 2. Main Text

### 2.1. Typical and Atypical Duplications

The *int22h1/int22h2*-mediated Xq28 duplication syndrome arises from duplications of the segment that extends from intron 22 homologous region 1 (*int22h1*) to intron 22 homologous region 2 (*int22h2*) within the q28 subregion of the X chromosome [15]. At the time of discovery of the syndrome, the duplication was difficult to detect using traditional chromosomal microarray analysis (CMA). However, with the improvement in resolution of the available CMA techniques, it has now become somewhat easier to detect the presence of the duplication with the majority of the available CMA techniques that are usually requested in the workup of intellectual disability and/or neurodevelopmental delay [15].

**Figure 1:**
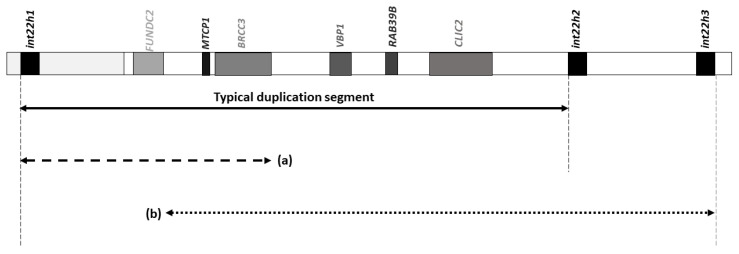
Schematic illustration of the duplicated chromosomal segment in *int22h1/int22h2*-mediated Xq28 duplication syndrome. The figure highlights the classical duplication detected in most patients diagnosed to date with the syndrome, as well as two atypical duplications recently detected: (**a**) a shorter duplicated segment that spans only the centromeric half of the typical duplication (~0.26 Mb), identified in a male subject, and (**b**) a 0.2 Mb-telomerically shifted duplication extending beyond the *int22h2* locus, detected in two siblings.

The classical duplication that was first described in affected individuals is approximately 0.5 Mb in size, which roughly corresponds to the size of the segment extending between *int22h1* and *int22h2* [15]. Recently, however, two ‘atypical’ versions of the duplication associated with the development of *int22h1/int22h2*-mediated Xq28 duplication syndrome have been reported [10] (Figure 1). The first is a shortened version of the typical duplication, measuring approximately 0.26 Mb and spanning only the centromeric half of the segment involved in the typical duplication, such that the distal breakpoint of this duplication falls centromeric to *int22h2*, between *int22h1* and *int22h2* [10] (Figure 1a). In contrast, the second atypical duplication that has been recently reported in association with *int22h1/int22h2*-mediated Xq28 duplication syndrome, is a 0.2 Mb-telomerically shifted and slightly shortened (by approximately 0.1 Mb) version of the classical duplication, with its corresponding distal breakpoint lying telomeric to *int22h2* and within the intron 22 homologous region 3 (*int22h3*) [10] (Figure 1b). Most individuals reported to date with the duplication were found to have inherited their duplication from their respective mothers (i.e., maternally inherited duplication) [15]. More recently, however, two cases have been reported in which the duplication could not be detected in either parent of the respective proband, suggesting they were de novo duplications [10].

### 2.2. Common Clinical Features

The main clinical manifestations of *int22h1/int22h2*-mediated Xq28 duplication syndrome are intellectual disability, neurobehavioral abnormalities, and nonspecific facial dysmorphic features [15]. As mentioned earlier, however, due to the X-linked nature of the syndrome, affected males tend to exhibit more severe and pervasive features of the syndrome, compared with heterozygous females [15]. For instance, all affected males reported to date who could undergo formal neurocognitive assessment were found to have mild or moderate intellectual disability. In contrast, less than half of the heterozygous females reported to date were found to have intellectual disability, with the latter being mild only [15]. In fact, most heterozygous females are unaffected, with the remainder of them displaying little to no discernible cognitive deficits (Table 1). Likewise, affected males tend to exhibit a variety of neurobehavioral manifestations, namely aggressiveness, irritability, attention-deficit hyperactivity disorder (ADHD), impulsiveness, anxiety, apparent socialization deficits, and autism spectrum disorders (ASD) [15]. In contrast, most heterozygous females reported to date with neurobehavioral abnormalities exhibited behaviors consistent with those of the inattentive-type childhood ADHD, i.e., impulsiveness, inattention, and emotional lability, as well as mild to moderate socialization deficits in two females. Moreover, heterozygous females with neurobehavioral manifestations appear to present at a later age compared with affected males [15]. As for the nonspecific facial features associated with the syndrome, they are somewhat similar between affected males and heterozygous females, and include a tall forehead, sparse scalp hair, sparse eyebrows, depressed and highly seated nasal bridge, thin upper lip, thick lower lip, and large ears [15] (Table 1).

### 2.3. Speculated Molecular Basis of the Syndrome

It remains unclear to date which of the six genes located within the duplicated segment (i.e., *FUNDC2*, *MTCP1*, *BRCC3*, *VBP1*, *RAB39B*, and *CLIC2*) contribute(s) most to the intellectual disability and neurobehavioral manifestations associated with the *int22h1/int22h2*-mediated Xq28 duplication syndrome and whether these manifestations are monogenic or polygenic. Two genes, however, have been speculated to contribute the most to the syndrome’s cognitive and neurobehavioral features. These include *RAB39B* and *CLIC2*. The reason underlying such a speculation was the consistent detection of both genes within the smallest region of overlap between all individuals reported to date with the syndrome [10,17,18]. Interestingly, dosage alterations in each of these two genes have been previously reported to result in distinct forms of XLIDS [19,20,21]; copy number gains or alternatively, loss-of-function mutations in *RAB39B* have been associated with an XLIDS, known as X-linked mental retardation-72 (MRX72), which is characterized by macrocephaly, epilepsy, and ASD (OMIM# 300271) [17,19,22,23]. On the other hand, deletions or mutations affecting *CLIC2* have been associated with a different XLIDS, known as X-linked syndromic mental retardation-32 (MRXS32), characterized by cardiac tachyarrhythmias, congestive heart failure, cardiomegaly, and seizures (OMIM# 300886) [21]. Intriguingly, both of these distinct syndromes exhibit multiple overlapping features with int22h1/int22h2-mediated Xq28 duplication syndrome, namely intellectual disability, neurodevelopmental delays, ASD, hyperactivity, large ears, and kyphoscoliosis [10,17].

### 2.4. Sex-Specific Differences

However, besides the aforementioned main clinical features of the syndrome, affected males also exhibit two additional findings, not yet detected in any of the heterozygous females reported to date [15]; the first being obesity with or without a concomitant tall stature, and the second being an intriguing combination of recurrent sinopulmonary infections (i.e., sinusitis, recurrent upper respiratory tract infections, pneumonia, bronchitis, and otitis media) and atopic diseases (i.e., asthma, allergic rhinitis, and eczema) [15]. However, why only affected males simultaneously exhibit two extremes of immune dysregulation, i.e., atopy and recurrent infections, which mark a hyperactive immune response and immunodeficiency, respectively, remains unknown. Nonetheless, such sex-specific differences in immune responses are worth probing, given the potential that understanding the immunological roles of the segment duplicated in the syndrome may lend to our understanding of known differences in immune tolerance between males and females in general. It has been reported that females, via their second X chromosome, possess an “immunological advantage” and a stronger immune response against pathogens, compared to males, which may explain why females appear to be less likely to have immunodeficiency than males [24,25]. At the same time, such differences paradoxically impart a disadvantage to females with regards to their increased likelihood of developing autoimmune diseases [24,25]. Thus, it is rather intriguing in this syndrome that only males carrying the duplication seem to simultaneously exhibit both extremes of the immune regulation spectrum. This warrants further investigation, with special emphasis on identifying the gene or genes, included in the duplication segment, that also possess immunomodulatory roles. The most strikingly possible candidate gene with such function appears to be *MTCP1*, which falls within the duplicated segment and encodes a leukemogenic oncoprotein previously implicated in the survival and proliferation of mature T lymphocytes [26]. More interestingly, translocations involving *MTCP1* have also been identified in a subset of patients with ataxia telangiectasia, which is known for its association with both immunodeficiency manifesting as recurrent sinopulmonary infections, and increased cancer risk [27]. Both of these features have been reported in male individuals with the *int22h1/int22h2*-mediated Xq28 duplication syndrome [10]. Nonetheless, whether *int22h1/int22h2*-mediated Xq28 duplication-associated *MTCP1* dosage alterations underlie the peculiar immune manifestations seen exclusively in males with the *int22h1/int22h2*-mediated Xq28 duplication syndrome, remains uninvestigated.

Another finding associated with the syndrome that intriguingly manifests differently in affected males versus heterozygous females is insomnia, more specifically, the type of sleep disturbance experienced by each; while sleep disturbances seem to occur at nearly equal frequencies among the two sexes, all three affected males reported to date with sleep disturbances were found to have difficulty remaining asleep during the night, compared with the two heterozygous females reported to date with sleep disturbances that had difficulty falling asleep (i.e., defect in sleep maintenance versus sleep initiation, respectively) [10] (Table 1).

### 2.5. Rare Malformations and Less Common Features

Several additional infrequent manifestations have been reported in association with the syndrome, including genitourinary malformations (e.g., cryptorchidism, hydronephrosis, and hypospadias), imperforate anus, limb and digital anomalies (e.g., clinodactyly, rocker-bottom feet, arthrogryposis, radial polydactyly, etc.), spinal anomalies (e.g., kyphoscoliosis, sacral agenesis, and missing ribs), cardiac anomalies (e.g., atrial septal defect, patent ductus arteriosus, etc.), micrognathia, motor mannerisms, strabismus, hearing loss, hypotonia, hemihypertrophy, cleft lip and cleft palate, and esophageal atresia with trachea-esophageal fistula [15] (Table 1). Two recently reported findings, in particular, multiple malignancies and cleft lip and cleft palate, are worth highlighting, given their potential implications for other medical fields [10]. Of the 35 individuals reported to date with *int22h1/int22h2*-mediated Xq28 duplication syndrome, a recently reported affected male was found to carry the duplication after he underwent extensive genetic workup to investigate his history of multiple cancers [10]. The patient tested negative for any of the genes associated with hereditary and genetic cancer syndromes commonly screened for. However, whether his history of multiple cancers is simply a coincidental finding unrelated to his duplication, remains to be probed. Nonetheless, among the two genes suspected to contribute most to the syndrome’s neurobehavioral manifestations, *RAB39B* and *CLIC2*, the former has been shown to act as an oncogene and has been previously associated with multiple types of cancer [19,28], while the latter has also been shown to be altered in various types of tumors [29,30]. Alternatively, another gene that lies centromeric to the latter genes within the duplicated segment, *VBP1*, has also been raised as a potential suspect possibly underlying that patient’s development of multiple tumors. Such a speculation was based on a combination of facts pertaining to *VBP1*: it encodes a tumor-suppressor protein involved in repairing DNA damage, has been associated with the development of multiple cancers, and is not included in the gene panels commonly used to screen for hereditary or genetic cancers [10,31,32].

Besides the single case of the syndrome with a history of multiple malignancies, two affected males have been reported to date to have cleft lip and cleft palate [15]. However, while the first male carried the typical 0.5 Mb duplication, the second one with cleft lip and cleft palate was the one with the atypical, shortened version of the duplication previously mentioned [10]. Through aligning the coordinates of both duplications, the authors reporting the case argued that, since the smallest region of overlap between the classical and this newly detected atypical duplication only includes the *BRCC3* locus, the latter is likely the culprit for the development of cleft lip and cleft palate [10]. Interestingly, it has been previously reported twice that certain non-syndromic forms of cleft lip and cleft palate are linked to alterations in the *BRCA1* and *BRCA2* genes, whose products interact directly with BRCC3 in mediating their physiological effects [33,34]. The association of each of these two recently reported ‘unique’ findings in association with the syndrome, i.e., multiple malignancies and cleft lip and cleft palate, remain to be confirmed. However, the value of highlighting these observations lies in the hope that it will prompt research into further understanding the role of the genes included in the duplicated segment, in carcinogenesis and facial morphogenesis, respectively.

## 3. Conclusions

To summarize, the *int22h1/int22h2*-mediated Xq28 duplication syndrome is an ultra-rare XLIDS arising from duplications involving the subregion flanked by *int22h1* and *int22h2* on Xq28. The main clinical manifestations of this syndrome include intellectual disability, neurobehavioral abnormalities, and facial dysmorphia. However, due to the X-linked nature of the syndrome, affected males generally show more severe phenotypes compared to heterozygous females, who may be completely asymptomatic. Apart from the three aforementioned manifestations of the syndrome, a unique combination of atopy and recurrent infections has been noted in a large proportion of affected males reported to date. Such a feature has not been seen in any of the heterozygous females identified to date with this syndrome. Likewise, another sex-variable manifestation noted for the syndrome is the nature of insomnia when present. Specifically, while affected males appear to predominantly have difficulty maintaining sleep (i.e., sleep-maintenance insomnia), heterozygous females primarily have difficulty initiating sleep (i.e., sleep-onset insomnia). Finally, two unique findings recently reported in association with the syndrome that warrant highlighting are multiple malignancies, and cleft lip and/or palate. These latter findings possibly suggest key roles for the genes included in the duplicated segment, in tumorigenesis and facial morphogenesis, respectively. In particular, they highlight an increased gene dosage in *BRCC3* as a possible alteration underlying cleft lip and cleft palate development, warranting future probing of this locus in non-syndromic cleft lip or palate cases. Additionally, the finding of multiple malignancies in association with the syndrome, an observation recently replicated by our colleagues, raises a potential role for the genes within the duplicated region in regulating cellular growth and proliferation.

## Figures and Tables

**Table 1:** The common and sex-specific features of *int22h1/int22h2*-mediated Xq28 duplication syndrome.

		Males	Females
**Frequent Features**		Tall forehead Large ears Sparse scalp and eyebrow hair Thin upper lip Thick lower lip Long eyelashes High-rooted and depressed nasal bridge	
**Rare Features**		- Genitourinary malformations (hydronephrosis, cryptorchidism, hypospadias) - Digital and limb malformations (radial polydactyly, arthrogryposis, clubfoot, 2–3 toe syndactyly) - Hearing loss - Strabismus and/or myopia - Cleft lip and/or cleft palate - Esophageal atresia with trachea-esophageal fistula - Polyhydramnios - Congenital heart disease (atrial septal defect with concomitant VSD or PDA) - Generalized hypotonia - Skeletal malformations (sacral agenesis, missing ribs, vertebral malformations, hip dysplasia) - Psychotic disorders - Multiple malignancies	- Recurrent seizures - Café-au-lait macule - Freckling - Hemihypertrophy - Hypothyroidism
		Clinodactyly Motor mannerisms and/or stereotypy Micrognathia kyphoscoliosis	
***Distinguishing Features***	*Intellectual disability*	Mild-to-moderate *(complete penetrance)*	Mild or none *(incomplete penetrance)*
	*Type of insomnia* *(if and when present)*	Difficulty maintaining sleep	Difficulty falling asleep
	*Anthropometric abnormalities*	Obesity +/− tall stature	Obesity reported in only one female to date
	*Immune manifestations*	Recurrent sinopulmonary infections + atopy	Neither
	*Neurobehavioral and psychiatric manifestations*	Aggression and irritability	**-**
		Predominantly hyperactive-type childhood ADHD	Predominantly inattentive-type childhood ADHD
		Autism spectrum disorder	Mild-to-moderate socialization deficits

VSD: Ventricular septal defect; PDA: patent ductus arteriosus; ADHD: attention-deficit hyperactivity disorder. ‘**-**’ denotes the lack of the same or comparable feature in heterozygous females, compared to the corresponding feature in affected males.

## Data Availability

All data included in this review have been previously published and are available online. If further specific data is needed, it may be provided by the corresponding authors upon reasonable request, provided that sharing such data does not jeopardize patient confidentiality.

## List of Abbreviations

XLIDS	X-linked Intellectual Disability Syndrome
Xq28	Region q28 of the X Chromosome
Mb	Mega-bases (=1 million bases)
*int22h1*	Intron 22 Homologous Region 1
*int22h2*	Intron 22 Homologous Region 2
*int22h3*	Intron 22 Homologous Region 3
*RAB39B*	Ras-Related Protein Rab-39B
*CLIC2*	Chloride Intracellular Channel Protein 2
*VBP1*	Von Hippel-Lindau Binding Protein 1
*BRCC3*	E3 Ubiquitin Ligase Subunit of the BRCA1-BRCA2-Containing Complex
*MTCP1*	Mature T-Cell Proliferation-1
CMA	Chromosomal Microarray Analysis
ADHD	Attention-Deficit Hyperactivity Disorder
ASD	Autism Spectrum Disorder
DNA	Deoxyribonucleic Acid
